# The Critical Role of Phenylpropanoid Biosynthesis Pathway in Lily Resistance Against Gray Mold

**DOI:** 10.3390/ijms252011068

**Published:** 2024-10-15

**Authors:** Qi Cui, Xinran Li, Shanshan Hu, Dongfeng Yang, Ann Abozeid, Zongqi Yang, Junhao Jiang, Ziming Ren, Danqing Li, Dongze Li, Liqun Zheng, Anhua Qin

**Affiliations:** 1 Laboratory of Flower Bulbs, Department of Landscape Architecture, Zhejiang Sci-Tech University, Hangzhou 310018, China; hestia_1201@163.com (X.L.); hushanshan2024@163.com (S.H.); jiangjunhao@zstu.edu.cn (J.J.); zimingren@zju.edu.cn (Z.R.); danqingli@zju.edu.cn (D.L.); dongzeli06@163.com (D.L.); zlq@zstu.edu.cn (L.Z.); qah0208@163.com (A.Q.); 2 Key Laboratory of Plant Secondary Metabolism and Regulation of Zhejiang Province, College of Life Sciences and Medicine, Zhejiang Sci-Tech University, Hangzhou 310018, China; yangdongfeng@zstu.edu.cn (D.Y.); annabozeid@science.menfia.edu.eg (A.A.); yangzongqi@zstu.edu.cn (Z.Y.)

**Keywords:** lily, *Botrytis elliptica*, transcriptome, metabolome, phenylpropanoid, transcriptional regulatory

## Abstract

Gray mold caused by *Botrytis elliptica* is one of the most determinative factors of lily growth and has become a major threat to lily productivity. However, the nature of the lily *B. elliptica* interaction remains largely unknown. Here, comparative transcriptomic and metabolomic were used to investigate the defense responses of resistant (‘Sorbonne’) and susceptible (‘Tresor’) lily cultivars to *B. elliptica* infection at 24 hpi. In total, 1326 metabolites were identified in ‘Sorbonne’ and ‘Tresor’ after infection, including a large number of phenylpropanoids. Specifically, the accumulation of four phenylpropanes, including eriodictyol, hesperetin, ferulic acid, and sinapyl alcohol, was significantly upregulated in the *B. elliptica*-infected ‘Sorbonne’ compared with the infected ‘Tresor’, and these phenylpropanes could significantly inhibit *B. elliptica* growth. At the transcript level, higher expression levels of *F3′M*, *COMT*, and *CAD* led to a higher content of resistance-related phenylpropanes (eriodictyol, ferulic acid, and sinapyl alcohol) in ‘Sorbonne’ following *B. elliptica* infection. It can be assumed that these phenylpropanes cause the resistance difference between ‘Sorbonne’ and ‘Tresor’, and could be the potential marker metabolites for gray mold resistance in the lily. Further transcriptional regulatory network analysis suggested that members of the AP2/ERF, WRKY, Trihelix, and MADS-M-type families positively regulated the biosynthesis of resistance-related phenylpropanes. Additionally, the expression patterns of genes involved in phenylpropanoid biosynthesis were confirmed using qRT-PCR. Therefore, we speculate that the degree of gray mold resistance in the lily is closely related to the contents of phenylpropanes and the transcript levels of the genes in the phenylpropanoid biosynthesis pathway. Our results not only improve our understanding of the lily’s resistance mechanisms against *B. elliptica*, but also facilitate the genetic improvement of lily cultivars with gray mold resistance.

## 1. Introduction

Plants face numerous biotic stresses throughout their life cycles that have negative effects on their growth and development. Over time, plants have evolved complex and efficient immune mechanisms to combat the invasion of pathogens [1]. In response to pathogen attack, plant pattern recognition receptors located on the cell membrane play a key role in the recognition of pathogen-associated molecular patterns, and they trigger a series of downstream defense responses, which are closely correlated with quantitative resistance. On the other hand, pathogens release effector proteins to infect plants, however, plants produce resistance proteins encoded by resistance genes to counteract these effectors, leading to effector-triggered immunity, which is also known as qualitative resistance [2]. Quantitative resistance, which is controlled by multiple defense-related genes and secondary metabolites, is regarded as the important immune mechanism in plants [3].

The combination of transcriptomic and metabolomic analyses provides a powerful approach for obtaining a comprehensive insight into the mechanisms underlying quantitative resistance. For example, integrated transcriptomic and metabolomic analyses have revealed that phenylpropanoid biosynthesis plays a crucial role in the resistance of many plants to pathogenic fungi, such as the resistance of *Zea mays* to *Fusarium verticillioides* [4], *Brassica oleracea* to *Alternaria brassicicola* [5], and *Lilium regale* to *Fusarium oxysporum* [6]. Similarly, flavonoid biosynthesis was found to positively regulate the resistance of *Zanthoxylum bungeanum* defense against stem canker caused by *Fusarium zanthoxyli*. Further study demonstrated that plant hormones, such as gibberellin (GA), abscisic acid (ABA), ethylene (ET), brassinosteroids (BRs), jasmonic acid (JA), and salicylic acid (SA) were regulators of the flavonoid metabolism that activated the resistance in *Z*. *bungeanum* [7]. Additionally, the higher accumulation of phenylpropanoids and a-linolenic acid in the *Alternaria alternata*-resistant sweet cherry (*Prunus avium*) led to more lignin accumulation and the early induction of JA signaling, respectively, which enhanced the resistance to *A. alternata* [8]. Moreover, diterpenoid biosynthesis and thiamine metabolism were observed to play critical roles in the resistance of Secale cereale against leaf rust caused by *Puccinia recondita* [9]. Transcriptomics and metabolomics have also been used to study pathogen interactions with other plant hosts, including *Solanum lycopersicum* [10], *Ipomoea batatas* [11], *Piper nigrum* [12], and *Brassica napus* [13]; furthermore, many defense-related genes and metabolites were identified that revealed the molecular responses. Therefore, comprehensive transcriptomic and metabolomic analyses can provide a better understanding of plant defense against pathogens at the molecular level, which will accelerate the development and selection of disease resistant cultivars.

The lily (*Lilium* spp.), which is one of the most important ornamental plants in the world, can be used as both a cut-flower crop and a potted plant. However, the yield and quality of lilies face serious threats from diseases caused by various pathogens. Gray mold, which is mainly caused by *Botrytis elliptica*, is a prevalent and devastating lily fungal disease throughout the world [14]. It has been reported that *B. elliptica* primarily attacks the stem, leaf, and floral tissues of lilies by producing effectors that exclusively cause apoptotic cell death, leading to huge economic losses every year [15]. Growing resistant cultivars is a cost-effective and environmentally friendly way to manage lily gray mold disease. So far, the germplasm conferring complete resistance against *B. elliptica* has not been identified in the lily. Germplasm with limited resistance primarily exists in some wild species (*L. regale*) and cultivars from Oriental hybrids and Oriental × Trumpet hybrids [16]. Thus, the utilization of the resistance of the lily will help to obtain high-resistance germplasm and achieve green and safe control strategies for *B. elliptica*. The lily’s resistance to gray mold is a typical quantitative trait [17]. In recent years, several key genes involved in the responses to *B. elliptica* infection have been observed. These genes are involved in the SA/JA pathway, MAPK/Ca^2+^ pathway, secondary metabolism biosynthesis pathway, etc. Additionally, a group of genes encoding pathogenesis-related proteins (PRs), glycine-rich protein (GRP), and WRKY transcription factors has also been identified [18,19,20,21,22]. A new study has shown that the LlWRKY33-LlHSFA4-LlCAT2 regulatory module confers gray mold resistance in the lily via a reduction in cell death and ROS accumulation [23]. However, there are no studies on comparative transcriptomic and metabolomic analyses of resistant and susceptible lilies infected with *B. elliptica*, and some genes and metabolites that are closely associated with disease resistance remain unexplored.

In order to clarify the resistance mechanisms of the lily against gray mold, we combined transcriptomic and metabolomic analyses for the first time to study two lily cultivars with contrasting resistance to gray mold; furthermore, we characterized the differentially expressed genes (DEGs) and differentially accumulated metabolites (DAMs) involved in the defense response during *B. elliptica* infection. Important resistance-related genes, pathways, and metabolites were identified. The correlations between gene expression and metabolite biosynthesis were analyzed. Moreover, the results of the transcriptomic and metabolomic analyses were validated via quantitative real-time polymerase chain reaction (qRT-PCR) and antifungal experiments. This study offered valuable information regarding the defense mechanism of the lily against *B. elliptica* infection, which will greatly facilitate the genetic improvement of lily cultivars with high gray mold resistance.

## 2. Results

### 2.1. Leaf Phenotypes of Two Lily Cultivars after Infection by B. elliptica

**Figure 1:**
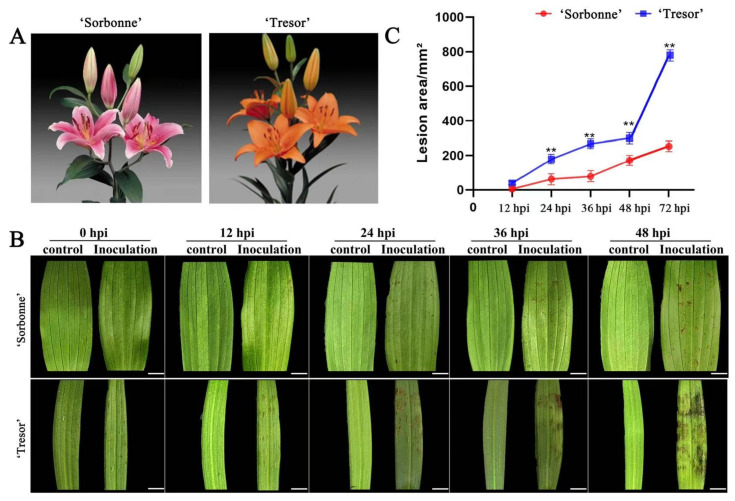
(**A**) Oriental hybrid ‘Sorbonne’ and Asiatic hybrid ‘Tresor’. (**B**) Leaf phenotype of ‘Sorbonne’ and ‘Tresor’ after infection with *B. elliptica*, scale bar = 1 cm. (**C**) Leaf lesion size over time of ‘Sorbonne’ and ‘Tresor’ after infection with *B. elliptica*, n = 12. The lesion areas are shown as the means of the three biological replicates ± SD. Asterisks indicate statistically significant differences between resistant and susceptible cultivars according to Student’s *t*-test (** *p* < 0.01).

A highly resistant Oriental hybrid, ‘Sorbonne’, and a highly susceptible Asiatic hybrid, ‘Tresor’, were selected based on our previous evaluation of different lily cultivars for resistance to *B. elliptica* [24] (Figure 1A). *B. elliptica* was inoculated on young, healthy leaves of ‘Sorbonne’ and ‘Tresor’. The infected leaves of both cultivars showed obvious symptoms of gray mold during the fungus infection. Twelve hours after inoculation, water-soaked symptoms initially appeared on the susceptible cultivar ‘Tresor’ around the inoculation sites, which later turned into light brown spots. With the development of the infection, these lesions subsequently developed into necroses and expanded rapidly until a darkening of the color occurred at the inoculation site after 72 h. In contrast, the lesion enlargement was limited, and only a few lesions were observed on the leaves of ‘Sorbonne’ (Figure 1B). The development of the lesion area was also measured. At 72 h after inoculation with *B. elliptica*, the lesion area of ‘Sorbonne’ was only 35.5% of that of ‘Tresor’ and was significantly smaller (at *p* < 0.01) than that of ‘Tresor’ throughout the infection process (Figure 1C).

### 2.2. Transcriptome Profile of Lily in Response to B. elliptica Infection

#### 2.2.1. Overview and Analysis of Transcriptome Sequencing Data

To analyze the transcriptomic differences between the resistant and susceptible lily leaves and to identify the disease resistance-related genes, young leaves from ‘Sorbonne’ and ‘Tresor’ were inoculated with *B. elliptica*. After inoculation for 24 h, healthy (uninoculated ‘Sorbonne’ and ‘Tresor’ were named RH and SH, respectively) and diseased (inoculated ‘Sorbonne’ and ‘Tresor’ were named RD and SD, respectively) leaves were collected for RNA-seq analysis. A total of 82.94 Gb of clean data were obtained. The numbers of clean reads for each sample ranged from 19,433,566 to 37,417,417, and the percentages of GC and Q30 were 47.72–49.60% and 97.05–97.55%, respectively (Table S1). After de novo assembling, 285,345 transcripts were generated, with a mean length of 940 bp and an N50 of 1252 bp. Finally, 122,373 unigenes were obtained, with a mean length of 850 bp and an N50 length of 1151 bp (Table S2). These unigenes were then annotated using the NCBI non-redundant protein (Nr) (42.84%), Swiss-Prot (30.40%), Protein family (Pfam) (29.51%), Gene Ontology (GO) (29.14%), NCBI non-redundant nucleotide sequences (Nt) (21.41%) and KEGG Orthology (KO) (15.25%) databases to predict the biological functions (Figure S1). The unigenes were aligned against those of other species; numerous unigenes were mapped to *Elaeis guineensis* (10.6%) and *Phoenix dactylifera* (10.1%). This result showed that lilies have the highest homology with these Arecaceae plants. A correlation matrix analysis showed that the biological duplication was satisfactory and suitable for the subsequent analysis (Figure S2).

#### 2.2.2. Identification and Functional Enrichment Analysis of DEGs in Response to *B. elliptica* Infection

**Figure 2:**
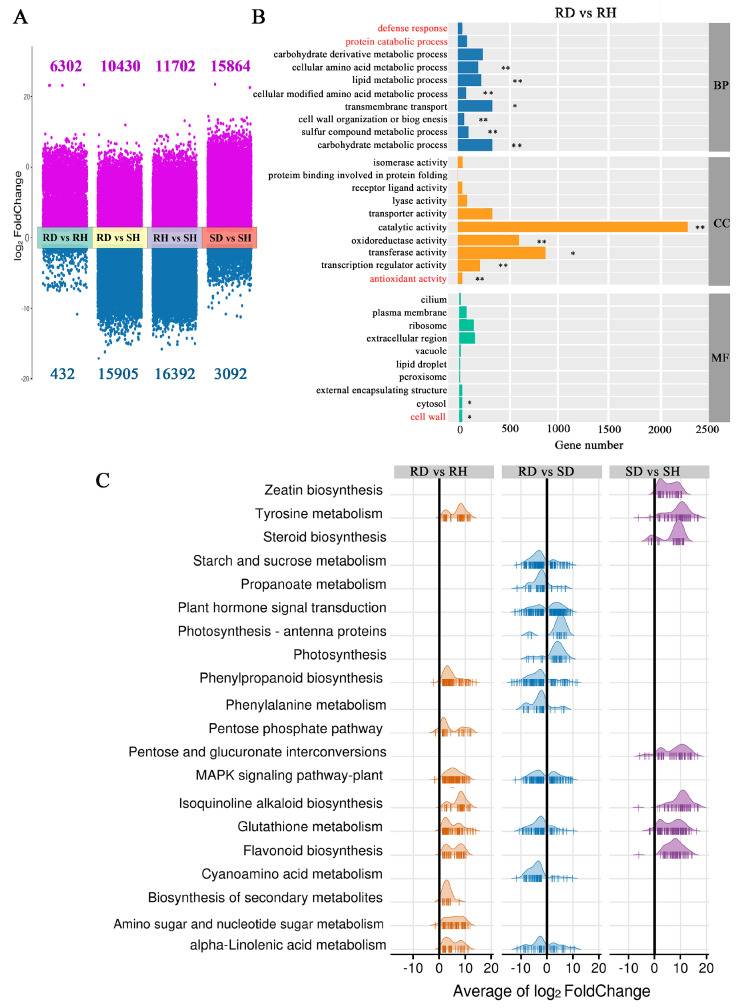
(**A**) Identification and functional enrichment analysis of DEGs in lily. (**A**) Volcanic plots of DEGs. Red and blue points represent the up-regulated and down-regulated DEGs, respectively. RD and SD represent *B. elliptica*-inoculated samples of ‘Sorbonne’ and ‘Tresor’, respectively. RH and SH represent the controls of ‘Sorbonne’ and ‘Tresor’, respectively. (**B**) GO enrichment analysis of the DEGs detected in *B. elliptica*-infected ‘Sorbonne’ compared with its control. Red texts represent four defense-related terms that were specifically enriched in ‘Sorbonne’ after infection relative to ‘Tresor’. * *p* < 0.05, ** *p* < 0.01. (**C**) The ridge plot of KEGG enrichment analysis for the DEGs. The average log_2_FoldChange value of each gene in the pathway is shown in the ridge plot. If the value is greater than 0, the gene is the upregulated (right), and the gene is downregulated (left) if its value is less than 0. Three biological replicates were used for transcriptomic analysis.

DESeq 2 software was used to analyze the expression of the DEGs. Compared with their respective controls, 6734 DEGs (6302 up-regulated and 432 down-regulated) were identified from ‘Sorbonne’, and 18,956 DEGs (15,864 up-regulated and 3092 down-regulated) were identified from ‘Tresor’ after infection with *B. elliptica*. Additionally, a total of 26,335 DEGs (10,430 up-regulated and 15,905 down-regulated) were screened between ‘Sorbonne’ and ‘Tresor’ after infection (Figure 2A).

To further clarify the gene function involved in the *B. elliptica* response, the DEGs were analyzed using the GO and KEGG databases. The GO enrichment analysis showed that four defense-related terms were specifically enriched in ‘Sorbonne’ after infection relative to ‘Tresor’, including protein catabolic process, defense response, antioxidant activity, and cell wall (Figure 2B and Figure S3). The KEGG enrichment analysis showed that four pathways (tyrosine metabolism, isoquinoline alkaloid biosynthesis, glutathione metabolism, and flavonoid biosynthesis) were significantly enriched in both ‘Sorbonne’ and ‘Tresor’ after infection, indicating that these pathways are the common defense responses of lilies to *B. elliptica* (Figure 2C). Furthermore, there were six pathways specifically enriched in ‘Sorbonne’ after infection, including the phenylpropanoid biosynthesis and biosynthesis of secondary metabolites (gensenoside, mugineic acid, benzoxazinoid, hordatine, etc.), and the MAPK signaling pathway-plant, alpha-linolenic acid metabolism and carbohydrate metabolism (pentose phosphate pathway, amino sugar, and nucleotide sugar metabolism). Most of the DEGs enriched in these pathways were significantly upregulated in *B. elliptica*-infected ‘Sorbonne’ compared with its control. On the other hand, three pathways (biosynthesis of zeatin and steroid, pentose, and glucuronate interconversions) were specifically enriched in ‘Tresor’ after infection. Additionally, seven pathways were specifically enriched in *B. elliptica*-infected ‘Sorbonne’ relative to ‘Tresor’ and included carbohydrate metabolism (starch and sucrose metabolism), amino acid metabolism (cyanoamino acid and phenylalanine metabolism), plant hormone signal transduction (ABA, JA and SA), photosynthesis, photosynthesis-antenna proteins, and propanoate metabolism. These results suggested that ‘Sorbonne’ and ‘Tresor’ show contrasting defense responses to *B. elliptica* infection by regulating different metabolic pathways. ‘Sorbonne’ coped with *B. elliptica* by regulating the biosynthesis of secondary metabolites, plant hormone signal transduction, the MAPK signaling pathway, amino acid and carbohydrate metabolism, and photosynthesis. However, the defense responses of ‘Tresor’ mainly involved the biosynthesis of zeatin and steroid, and pentose and glucuronate interconversions.

### 2.3. Characteristics of the Metabolome of Lily in Response to B. elliptica Infection

#### 2.3.1. Quality Control of Metabolomic Data

**Figure 3:**
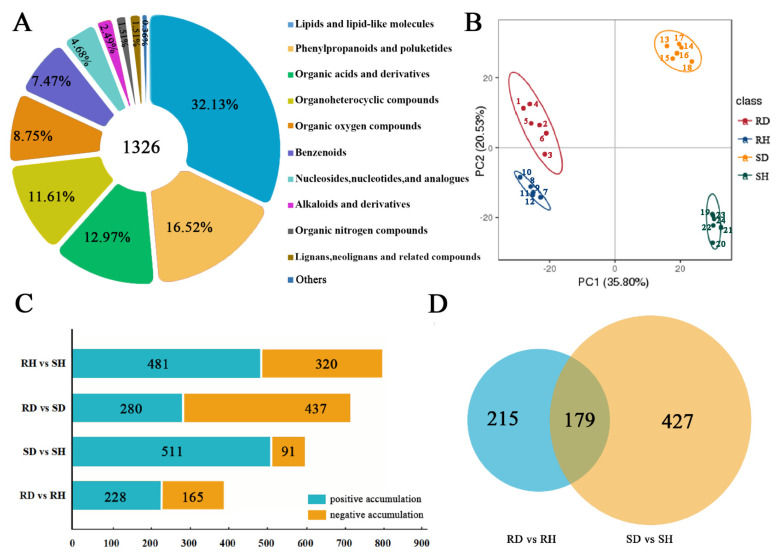
Classification of metabolites and identification of DAMs in ‘Sorbonne’ and ‘Tresor’ after infection with *B. elliptica*. (**A**) Classification and composition of identified metabolites. (**B**) Principal component analysis (PCA) of the samples based on the identified metabolites. (**C**) Numbers of DAMs identified in different pairwise comparisons. (**D**) Venn diagram of DAMs identified in ‘Sorbonne’ and ‘Tresor’ after infection with *B. elliptica*. Six biological replicates were used for metabolomic analysis.

Metabolomic analysis was conducted to determine the differences in the metabolite accumulation in ‘Sorbonne’ and ‘Tresor’ after infection with *B. elliptica*. A total of 1326 metabolites were identified and could be divided into 15 classes. Among them, phenylpropanoids and polyketides accounted for 16.52% of the total identified metabolites, making them second only to the lipids and lipid-like molecules (32.13%) in number (Figure 3A, Table S3). Principal component analysis (PCA) of 24 samples was conducted; PC1 accounted for 39.80% of the variability; fewer variations were observed among the biological replicates for each sample, but there were great variations between the samples of ‘Sorbonne’ and ‘Tresor’ based on the PCA (Figure 3B). In addition, partial least squares discriminant analysis (PLS-DA) was performed to analyze the differences between the samples; ‘Sorbonne’ and ‘Tresor’ also showed a relatively large separation (Figure S4). 

#### 2.3.2. Identification and Functional Enrichment Analysis of DAMs in Response to *B. elliptica* Infection

A total of 393 DAMs (228 up-regulated and 165 down-regulated) were identified in ‘Sorbonne’ after *B. elliptica* infection (Figure 3C), and 602 DAMs (511 up-regulated and 91 down-regulated) were detected in ‘Tresor’. There were 179 DAMs commonly detected in ‘Sorbonne’ and ‘Tresor’ after infection (Figure 3D). The KEGG enrichment analysis of the common DAMs showed they were enriched in many pathways, including tropane, piperidine and pyridine alkaloid, isoflavonoid, and terpenoid backbone biosynthesis pathways, and vitamin B6 and phenylalanine metabolism pathways, indicating that they are involved in the defense responses to *B. elliptica* (Figure S5). Nearly, all of the 179 DAMs were highly accumulated in ‘Sorbonne’ and ‘Tresor’ after infection with *B. elliptica*; only one DAM (methionine) showed decreased accumulation patterns in both cultivars (Table S4). 

**Figure 4:**
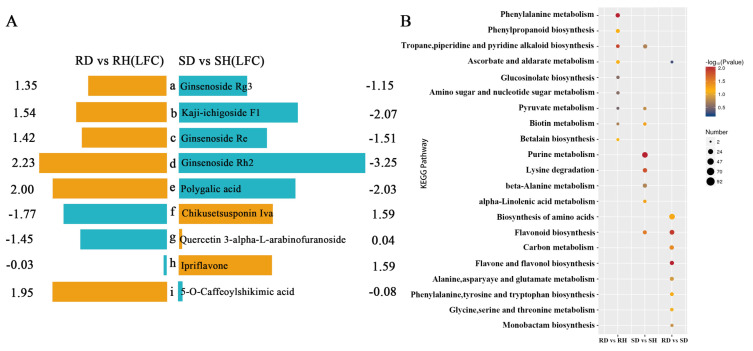
KEGG enrichment analysis of DAMs and in-depth analysis of key metabolites. (**A**) Six DAMs (a–f) with opposite accumulation patterns in ‘Sorbonne’ and ‘Tresor’ after infection with *B. elliptica* and three DAMs (g–i) were particularly accumulated in ‘Sorbonne’ or ‘Tresor’ after infection with *B. elliptica*. The horizontal axis indicates the value of log_2_FoldChange. Orange bars represent up-regulated DAMs, and blue bars represent the down-regulated DAMs. (**B**) KEGG enrichment analysis for DAMs.

The analysis of the DAMs indicated that numerous metabolites showed completely opposite accumulation patterns in ‘Sorbonne’ and ‘Tresor’ (Figure 4A). Terpenoids were reported to be involved in plant defense responses [25]. The accumulation of several triterpenes, including ginsenoside, kaji-ichigoside F1, and polygalic acid increased in resistant ‘Sorbonne’ while they decreased in susceptible ‘Tresor’ after *B. elliptica* infection. An opposite accumulation pattern was found for chikusetsusponin iva. These results suggested that chikusetsusponin iva was probably not directly involved in the *B. elliptica* resistance. However, ginsenoside, kaji-ichigoside F1, and polygalic acid might be closely related to the defense response of ‘Sorbonne’ against *B. elliptica*. In addition, several phenylpropanoids were observed to be specifically accumulated in ‘Sorbonne’ or ‘Tresor’ after *B. elliptica* infection (Figure 4A). For example, 5-O-caffeoylshikimic acid was specifically upregulated in ‘Sorbonne’, while quercetin 3-alpha-L-arabinofuranoside was specifically downregulated. Moreover, ipriflavone was specifically upregulated in ‘Tresor’. These results suggest that *B. elliptica* infection induces significant changes in different resistant cultivars and in the content of phenylpropanoids in ‘Sorbonne’ and ‘Tresor’. 

The KEGG enrichment analysis of the DAMs showed that three pathways were enriched in both ‘Sorbonne’ and ‘Tresor’ after infection, including tropane, piperidine, and pyridine alkaloid biosynthesis, pyruvate metabolism, and biotin metabolism, suggesting that they are involved in the common defense responses of lilies to *B. elliptica*. There were six pathways specifically enriched in ‘Sorbonne’ after infection, including phenylalanine metabolism, ascorbate and aldarate metabolism, amino sugar and nucleotide sugar metabolism, betalain biosynthesis, phenylpropanoid biosynthesis, and glucosinolate biosynthesis. On the other hand, five pathways were specifically enriched in ‘Tresor’ after infection, including purine metabolism, alpha-linolenic acid metabolism, beta-alanine metabolism, flavonoid biosynthesis, and lysine degradation (Figure 4B). These results demonstrated that ‘Sorbonne’ and ‘Tresor’ exhibit contrasting defense responses to *B. elliptica* by regulating different metabolic pathways.

### 2.4. Integrated Analysis of Transcriptomic and Metabolomic Data Revealed the Importance of Phenylpropanoid Biosynthesis for Lily Resistance to B. elliptica

**Figure 5:**
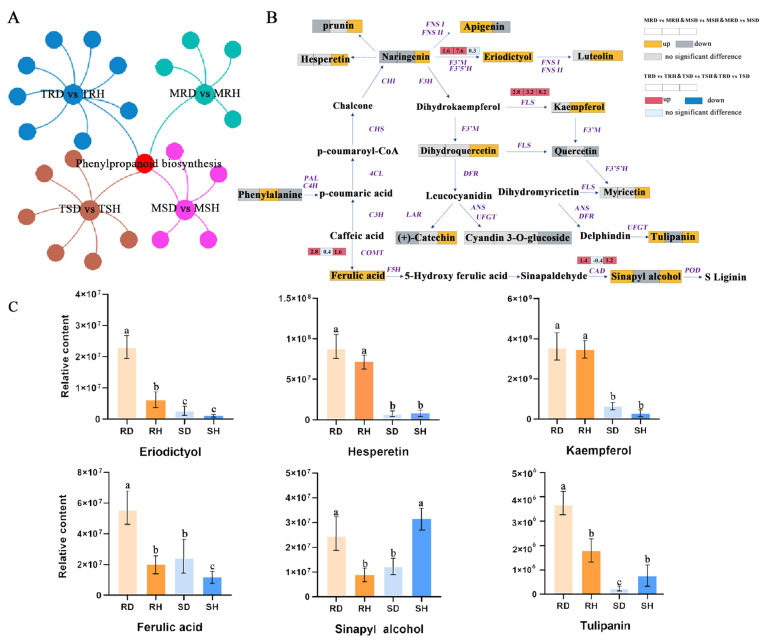
Integrated analysis of transcriptome and metabolome. (**A**) Venn diagram of KEGG pathways enriched in DEGs and DAMs in ‘Sorbonne’ and ‘Tresor’. MRD and MSD represent *B. elliptica*-inoculated samples of ‘Sorbonne’ and ‘Tresor’ metabolomes, respectively. MRH and MSH represent the controls of ‘Sorbonne’ and ‘Tresor’ metabolomes, respectively. TRD and TSD represent *B. elliptica*-inoculated samples of ‘Sorbonne’ and ‘Tresor’ transcriptomes, respectively. TRH and TSH represent the controls of ‘Sorbonne’ and ‘Tresor’ transcriptomes, respectively. (**B**) Diagram of partial phenylpropanoid biosynthesis pathway. The values of log_2_FoldChange for key DEGs are marked. (**C**) Comparison of the metabolite relative contents (represented as peak areas) between control and *B. elliptica*-infected ‘Sorbonne’ and ‘Tresor’. Data are presented as the means of the three biological replicates ± SD. Different letters above the bars indicate significant differences between treatments or genotypes according to Duncan’s multiple range test (*p* < 0.05).

To systematically understand the defense mechanisms of the lily against *B. elliptica* at both the transcriptomic and metabolic levels, integrated transcriptomic and metabolomic analyses were conducted. In total, ten and five KEGG pathways were significantly enriched in ‘Sorbonne’ after infection, respectively, based on the transcriptomic and metabolomic analyses (Figure 5A). However, only phenylpropanoid biosynthesis was commonly enriched for both the DEGs and DAMs in ‘Sorbonne’ after infection. Similarly, seven and five KEGG pathways were enriched in total for the DEGs and DAMs in ‘Tresor’ after infection, respectively (Figure 5A). Flavonoid biosynthesis, which is a branch of phenylpropanoid biosynthesis, was commonly enriched in the transcriptome and metabolome in ‘Tresor’. These results indicated the importance of phenylpropanoids in the lily’s resistance to *B. elliptica*. 

The profiles of the DAMs and DEGs in the phenylpropanoid pathway were further analyzed (Figure 5B,C). Most of the DAMs were involved in the lignin and flavonoid biosynthesis pathways. Among them, the accumulation of eriodictyol, kaempferol, and ferulic acid was increased in both ‘Sorbonne’ and ‘Tresor’ after infection with *B. elliptica*. They also showed higher contents in *B. elliptica*-infected ‘Sorbonne’ compared with ‘Tresor’. At the transcript level, an up-regulation of genes encoding flavonoid 3′-monooxygenase (F3′M) (Cluster-38572.30260), flavonol synthase (FLS) (Cluster-38572.42719), and caffeic acid 3-O-methyltransferase (COMT) (Cluster-38572.60593) were observed in the *B. elliptica*-infected ‘Sorbonne’ and ‘Tresor’ compared with their respective control, which might contribute to the accumulation of eriodictyol, kaempferol, and ferulic acid. Moreover, the accumulation of hesperetin, tulipanin, and sinapyl alcohol was significantly increased in ‘Sorbonne’, while it was decreased in ‘Tresor’ after infection with *B. elliptica*. At the transcript level, only one gene encoding cinnamyl alcohol dehydrogenase (CAD) (Cluster-38572.41309), which was upregulated in ‘Sorbonne’ and decreased in ‘Tresor’, was detected; thus, it might lead to the changes in the sinapyl alcohol content. Finally, the six critical DAMs mentioned above (eriodictyol, kaempferol, ferulic acid, hesperetin, tulipanin, and sinapyl alcohol) may play a critical role in the lily’s defense against *B. elliptica* and may cause the resistance differences between resistant and susceptible cultivars. Additionally, a discrepancy between the gene expression changes and their related metabolite accumulation patterns was found in the transcriptomic and metabolomic data, indicating that post-transcription regulation is widely involved in the lily’s defense against *B. elliptica*.

### 2.5. Transcriptional Network Regulating Phenylpropanoid Biosynthesis during Lily Defense Response to B. elliptica

**Figure 6:**
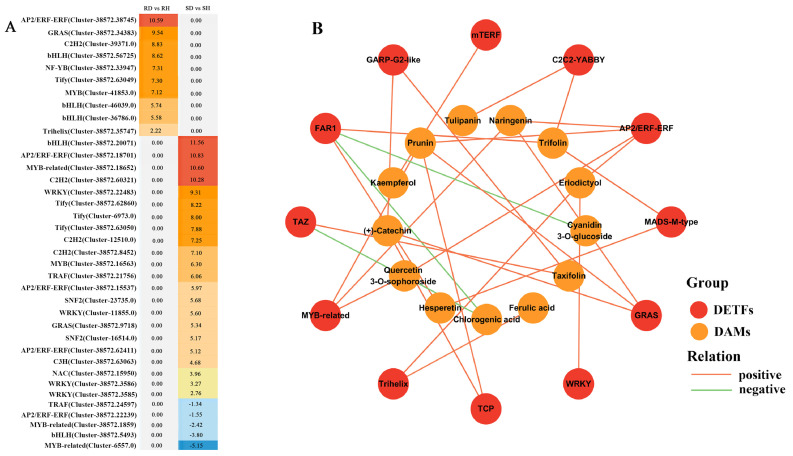
Transcription regulatory network analysis of phenylpropanes in lily after infection with *B. elliptica*. (**A**) Specifically expressed DETFs in ‘Sorbonne’ or ‘Tresor’. The values of log_2_FoldChange for DETFs are marked. (**B**) Transcription regulator network of phenylpropanes according to integrated analysis of transcriptome and metabolome. Red and orange circles represent the DETFs and DAMs, respectively.

Transcription factors (TFs) play an important regulatory role in plant defense responses. A total of 2294 unigenes were identified as TFs in this study, with 409 and 805 TFs found to be differentially expressed in ‘Sorbonne’ and ‘Tresor’ after infection with *B. elliptica*, respectively. The majority of differentially expressed TFs (DETFs) were distributed in the C2H2, WRKY, AP2/ERF, and bHLH families. Further analysis showed that many DETFs were found to be specifically expressed in ‘Sorbonne’ and ‘Tresor’ after *B. elliptica* infection (Figure 6A). Among them, 10 DETFs, which belonged to eight families, were significantly up-regulated in ‘Sorbonne’. A *AP2/ERF* gene (Cluster-38572.38745) showed the greatest changes in expression, with a log_2_ (Fold Change) value of 10.59. Moreover, there were 28 DETFs (22 up-regulated and 6 down-regulated) that were specifically expressed in ‘Tresor’. Among them, the AP2/ERF, MYB, and WRKY families were the most abundant. These results suggested that *B. elliptica* infection induces significant changes in transcriptional regulation in the resistant and susceptible cultivars. Those specially up-regulated TFs in ‘Sorbonne’ also have the potential to be used as marker genes for *B. elliptica* resistance in lilies. 

To explore the transcriptional regulation of phenylpropanoid biosynthesis, the correlation between the phenylpropane contents and expression patterns of the DETFs was established. Twelve kinds of TF families were detected to regulate 13 phenylpropanes (Figure 6B). Among them, the AP2/ERF and FAR1 family members were the most abundant, and both of them could regulate the biosynthesis of four phenylpropanes, with a Pearson correlation coefficient (rho) > 0.9. Except for the members of the FAR1 (Cluster-38775.0, Cluster-38572.33234) and TAZ (Cluster-41723.0) families, which were found to negatively regulate the biosynthesis of chlorogenic acid and cyanidin 3-O-glucoside (rho < −0.8), respectively, the majority of the DETFs showed a positive correlation with the phenylpropane contents. For example, the AP2/ERF (Cluster-38572.38745), WRKY (Cluster-46637.0), and Trihelix (Cluster-38572.35747) families were discovered to positively regulate the biosynthesis of eriodictyol. The Trihelix member (Cluster-38572.35747) was predicted to modulate ferulic acid biosynthesis. Additionally, the C2C2-YABBY (Cluster-38775.0), MADS-M-type (Cluster-38572.24041), and mTERF (Cluster-46623.0) families also played positive roles in the biosynthesis of tulipanin, hesperetin, and kaempferol, respectively (Table S5). 

### 2.6. qRT-PCR Validation of DEGs Associated with Phenylpropanoid Biosynthesis in Lily

**Figure 7:**
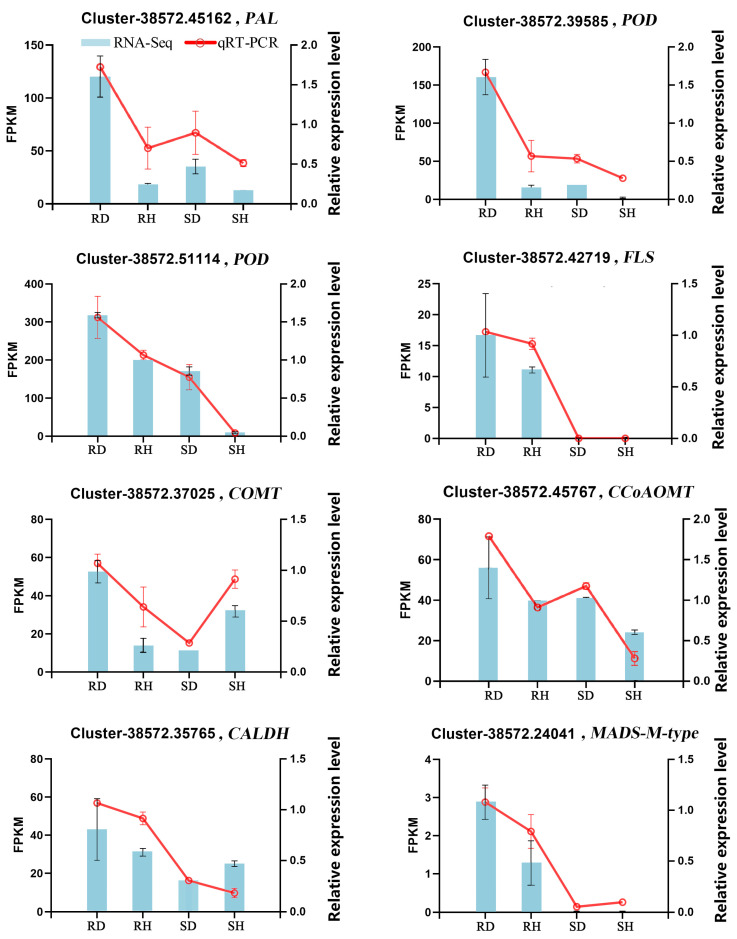
qRT-PCR validation of key candidate DEGs associated with phenylpropanoids metabolism in lily. The relative expression levels are shown as the means of the three biological replicates ± SD. RD and SD represent *B. elliptica*-inoculated transcriptomic samples of ‘Sorbonne’ and ‘Tresor’, respectively. RH and SH represent the controls of ‘Sorbonne’ and ‘Tresor’, respectively.

To validate the accuracy and repeatability of the transcriptomic analysis in the present research, qRT-PCR was conducted on a set of DEGs associated with phenylpropanoid metabolism. Among all the candidate DEGs related to phenylpropanoid biosynthesis, eight DEGs with higher expression levels in ‘Sorbonne’ than ‘Tresor’ after infection with *B. elliptica* were selected for qRT-PCR analysis. Figure 7 shows the expression levels of all the selected DEGs determined using qRT-PCR and RNA-Seq. On the whole, the expression patterns of the eight DEGs, as determined using qRT-PCR, were consistent with the corresponding FPKM values obtained using the RNA-Seq data. The critical genes for phenylpropanoid biosynthesis, i.e., *phenylalanine ammonia-lyase* (*PAL*), *peroxidase* (*POD*), *flavonol synthase* (*FLS*), *caffeic acid 3-O-methyltransferase* (*COMT*), *caffeoyl-CoA O-methyltransferase* (*CCoAOMT*), *coniferyl-aldehyde dehydrogenase* (*CALDH*), and *MADS-M-type*, were more highly expressed in ‘Sorbonne’ than in ‘Tresor’ after infection with *B. elliptica*. The majority of genes exhibited a similar expression profile using both methods. This result supported the reliability of the RNA-seq data. 

### 2.7. Phenylpropanes Inhibited B. elliptica Growth

**Figure 8:**
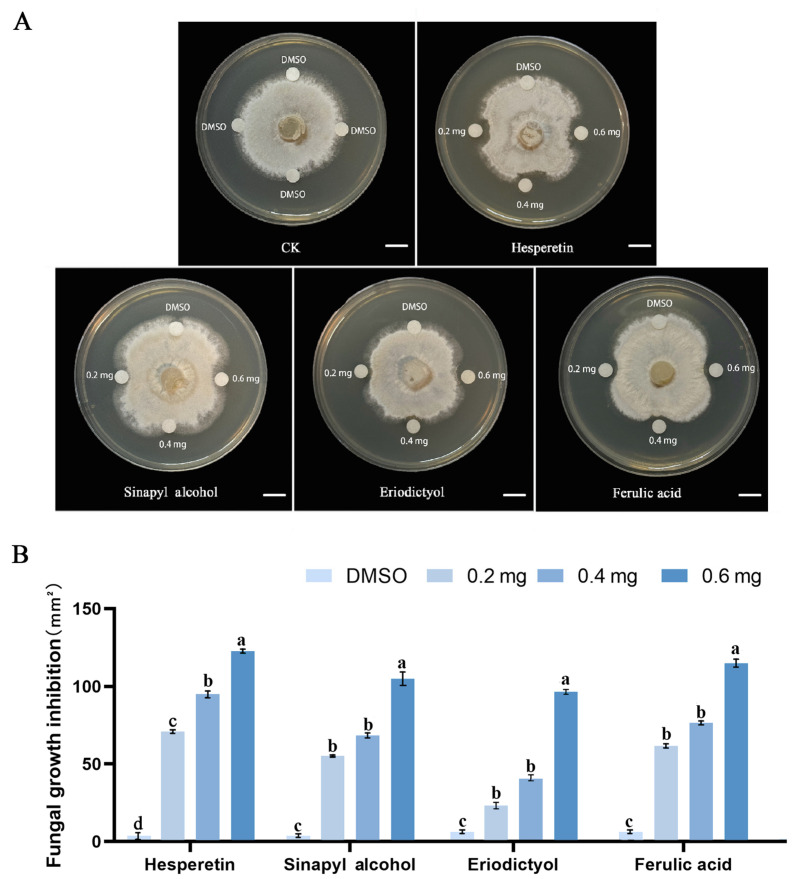
Inhibition effect of the phenylpropanes on *B. elliptica* growth. (**A**) Hesperetin, sinapyl alcohol, eriodictyol, and ferulic acid inhibited the growth of *B. elliptica*. DMSO was used as the solvent control. Scale bar = 1 cm. (**B**) The inhibition area of *B. elliptica* after treatment with hesperetin, sinapyl alcohol, eriodictyol, and ferulic acid, respectively. The inhibition areas are shown as the means of the three biological replicates ± SD. Different letters above the bars indicate significant differences among various treatments according to Duncan’s multiple range test (*p* < 0.01).

Based on the metabolomic data, several phenylpropanes were significantly accumulated in ‘Sorbonne’ compared with ‘Tresor’ after *B. elliptica* infection. Subsequently, plate inhibition assays of *B. elliptica* showed that the increasing concentrations of hesperetin, ferulic acid, eriodictyol, and sinapyl alcohol significantly inhibited the growth of *B. elliptica* (Figure 8A). Notably, 0.6 mg of hesperetin exhibited the most significant inhibitory effect, leading to a growth inhibition area of 121.4 mm^2^. Even at smaller concentrations (0.2 mg), hesperetin, ferulic acid, eriodictyol, and sinapyl alcohol could suppress *B. elliptica* mycelial growth, with growth inhibition areas of 70.3, 62.8, 21.7, and 54.8 mm^2^, respectively (Figure 8B). It can be assumed that the antifungal function of these phenylpropanes contributed to the resistance of ‘Sorbonne’ against *B. elliptica*.

## 3. Discussion

Gray mold is a prevalent fungal disease, not only for the lily but also for many other ornamental plants, such as *Paeonia lactiflora* [26], *Rose hybrida* [27], and *Cyclamen persicum* [28]. This disease could destroy 10–15% of the lilies on a farm after severe infection with *B. elliptica*, leading to serious economic loss [29]. Several gray mold-resistant resources, defense-related genes, and metabolites have been identified in lilies [19,23,24]. However, the interaction between the lily and *B. elliptica* and the defense mechanism are still far from understood, this has impeded genetic improvement of lily cultivars-to provide them with gray mold resistance. To address these problems, we performed comparative transcriptomic and metabolomic analyses to comprehensively reveal the defense differences between resistant and susceptible lily cultivars, thereby providing insights into the molecular basis of gray mold resistance. 

*Botrytis* species achieve plant cell death through the induction of apoptotic programmed cell death in the host, rather than killing the host cells directly [30]. A previous study indicated that *B. elliptica* produced host-specific effector that caused apoptotic cell death exclusively in the lily [31]. Host plants cope with the fungus attack via the innate immune responses initiated by a cognate receptor protein to recognize the effector and subsequently activate downstream components to trigger ROS burst, Ca^2+^ influx, MAPK signaling, plant hormone production, and transcriptional reprogramming [32]. Evidence is accumulating that plant MAPK cascades and plant hormone signal transduction play critical roles in plant resistance to pathogen attack [33,34,35]. In this study, the MAPK and plant hormone signaling (ABA, JA and SA) pathways were specifically enriched in *B. elliptica* infected ‘Sorbonne’ compared to ‘Tresor’, suggesting that they are both involved in the resistance of the lily to *B. elliptica* infection. Additionally, the GO enrichment analysis showed that antioxidant activity, cell wall organization, and defense response were significantly enriched in ‘Sorbonne’, suggesting the possibility that ‘Sorbonne’ inhibits *B. elliptica* invasion by antioxidant mechanisms for oxidative stress protection and preventing the fungus from injuring the cell wall.

Phenylpropanes can disrupt the plasma membrane of fungi, interfere with organelle functions, and inhibit cell wall formation, all of which have a suppression effect on the prevalence of fungal infections [36]. The phenylpropanoid metabolism-based defense responses towards pathogen attacks have been widely characterized in plants. In poplar, the anthracnose resistance is closely related to the types and contents of flavonoids and the transcript levels of the genes in the flavonoid biosynthesis pathway [37]. The application of certain phenylpropanes from *L. regale*, such as flavonoids, ferulic acid, phlorizin and quercetin, could restrict *F. oxysporum* growth [6]. Similarly, coumarin could enhance the resistance of sweet cherry (*Prunus avium*) to leaf spot disease [8]. In this study, the contents of four phenylpropanes, including eriodictyol, ferulic acid, hesperetin, and sinapyl alcohol in ‘Sorbonne’ were significantly higher than those in ‘Tresor’ after *B. elliptica* infection (Figure 5C), and they exhibited significant inhibitory effects on *B. elliptica* growth (Figure 8). It can be assumed that the antifungal function of these phenylpropanes contributes to the resistance of the lily to *B. elliptica* and that they can be used as metabolite markers for gray mold resistance in the lily. Interestingly, the expression changes in the *F3′M*, *COMT*, and *CAD* genes were consistent with the related metabolite accumulation patterns, which regulated the biosynthesis of the metabolites downstream (such as eriodictyol, ferulic acid, and sinapyl alcohol). Additionally, 5-O-caffeoylshikimic acid was found to be specifically accumulated in infected ‘Sorbonne’, while ipriflavone was specifically accumulated in infected ‘Tresor’ (Figure 4A). However, how these two phenylpropanes contribute to *B. elliptica* resistance is not known at present. The difference between them must be associated with the discrepancy in the *B. elliptica* resistance between ‘Sorbonne’ and ‘Tresor’. 

Transcription factors play pivotal roles in the accumulation of phenylpropanes during pathogen infections. Previous studies have demonstrated that OsMYB30 directly activates *4-coumarate:coenzyme A ligase* (*Os4CL*) genes, leading to increased lignin accumulation and the obvious thickening of sclerenchyma cells, inhibiting *Magnaporthe oryzae* penetration into *Oryza sativa* [38]. MdMYB1r1 positively regulates the *beta-glucosidase 40* (*MdBGLU40*) gene, resulting in high levels of coumarin and resistance to *Cytospora mali* in *Malus domestica* [39]. Recent research showed that the WRKY-MAPK regulatory module could promote GhMYC2-mediated flavonoid biosynthesis as a defense strategy against *Fusarium oxysporum* in *Gossypium hirsutum* [40]. In the current study, members of the AP2/ERF, WRKY, and Trihelix TF families were discovered to positively regulate the biosynthesis of eriodictyol. A Trihelix member was also predicted to modulate ferulic acid biosynthesis. Additionally, a member of the MADS-M-type family played a positive role in the biosynthesis of hesperetin (Figure 6B), implying that these TFs may have critical regulatory roles in the resistance of the lily against *B. elliptica*. The function of TFs in the lily’s disease resistance has not been thoroughly explored, and future work will focus on the in-depth analysis of these TFs to determine their roles. 

In addition to phenylpropanes, terpenoids also play important roles in plant disease resistance. JA-mediated terpenoid biosynthesis was reported to enhance resistance against *Alternaria alternata* in *Chrysanthemum morifolium* [25]. Multi-omics analysis has shown that a monoterpenoid (geranylgeranyl diphosphate) is involved in the resistance of *L. regale* to *F. oxysporum* [6]. Triterpenoid glycosides (prosapogenins) isolated from *Medicago sativa* were found to inhibit *Pyricularia oryzae* growth [41]. Furthermore, the accumulation of the triterpenoid withanolide could strengthen the resistance of *Withania somnifera* to diverse biotic stresses [42]. In this study, the contents of three kinds of triterpenoids (ginsenoside, kaji-ichigoside F1, and polygalic acid) were significantly increased in ‘Sorbonne’, while they were decreased in ‘Tresor’ after *B. elliptica* infection, demonstrating that these triterpenoids may play an important role during the interaction between the lily and *B. elliptica*. 

Integrated transcriptomic and metabolomic analyses offer a critical method for the mining of the metabolic networks and key genes involved in plant immunity [9,10]. In this study, the relationship between the DEGs and DAMs was established. It is worth noting that, in some cases, no significant correlations were detected between the accumulation patterns of the DAMs and the expression changes in the DEGs in the same enriched pathways, implying that posttranscription regulation widely existed during the *B. elliptica* infection. A similar phenomenon was also found in the defense responses of melons to gummy stem blight [43]. In addition, most metabolites came from the lilies, and a small portion of metabolites might come from *B. elliptica* in this study, the non-targeted metabolome we used could not distinguish the species sources of metabolites during this detection. However, when the KEGG analysis of DAMs was performed, it was species-specific, the DAMs in the KEGG pathway were all of plant origin, which could screened out some of the DAMs came from *B. elliptica*. Therefore, the DAMs on the phenylpropanoid biosynthesis pathway, which was the focus of this study, only came from the lilies. In summary, the current study offers new insights and valuable molecular information for understanding the gray mold resistance mechanism in the lily and will contribute to the selection of disease-resistant cultivars using marker-assisted breeding. However, the specific defense mechanism and relevant effector proteins still need to be investigated further. Therefore, other multiomics approaches, such as proteomic and genomic sequencing, should be used; with collaboration, a more comprehensive metabolic regulatory network for exploring the gray mold resistance in the lily can be built.

## 4. Materials and Methods

### 4.1. Lily Cultivation and B. elliptica Inoculation

The genus *Lilium* contains around 110 accepted species and thousands of polyploid cultivars, which can be classified in the different established hybrid groups (Asiatic, LA, OT, OA, LO, Longiflorum, and Oriental) [24]. Lilies can be propagated either by vegetative means or by sexual reproduction through seed production. Commercial potted and cut lily cultivars are considered perennial flowers; their foliage remains for only one growth season, and the bulbs are dormant in winter [44]. In this study, bulbs of the *Lilium* Oriental hybrid cv. ‘Sorbonne’ (resistant to *B. elliptica*) and Asian hybrid ‘Tresor’ (susceptible to *B. elliptica*) were planted in pots filled with substrate (sterile turf:vermiculite:perlite = 1:1:1, *v*/*v*/*v*) at 25/22 °C day/night temperatures with a 12/12 h photoperiod in a growth chamber in Zhejiang Sci-Tech University. *B. elliptica* (provided by the [Institute of Microbiology at the Chinese Academy of Sciences (Beijing, China)] was isolated from diseased lilies and grown on potato dextrose agar (PDA) medium under near-UV light for 7 days before use. Sampling of the lilies was performed during the flower bud stage. Detached leaves of ‘Sorbonne’ and ‘Tresor’ were inoculated with *B. elliptica*, according to the methods described previously [24]. Specifically, the procedure was as follows: *B. elliptica* mycelium disks (5 mm diameter) were collected and used to inoculate the detached lily leaves in vitro, while those uninoculated with *B. elliptica* served as the control. To maintain humidity (90–100%), all the leaves were placed in Petri dishes lined with moist filter paper, and the leaf petioles were wrapped with moist cotton. The detached leaves were sampled and photographed at 12, 24, 36, 48, and 72 h post-inoculation (hpi). The lesion areas were measured using the ImageJ version 2 software. Based on the phenotype of the disease lesions and the results of previous studies [19,24], the leaves were sampled at 24 hpi for transcriptomic and metabolomic sequencing. For transcriptomic analysis, three biological replicates were included, while six biological replicates were used for metabolomic analysis. 

### 4.2. Metabolomic Analysis

The extraction of metabolites was performed according to the method described previously [45]. In brief, the tissues were ground with liquid nitrogen, and the homogenate was resuspended with prechilled 80% methanol using a well vortex. The samples were incubated on ice for 5 min and then centrifuged. Some of the supernatant was diluted to a final concentration containing 53% methanol using LC-MS-grade water. Subsequently, the samples were transferred to a fresh Eppendorf tube and then centrifuged. Finally, the supernatant of the samples was injected into the ultra-performance liquid chromatography-tandem mass spectrometry (HPLC-MS/MS) system, and analysis was performed using a Vanquish UHPLC system coupled with an Orbitrap Q ExactiveTM HF mass spectrometer (Thermo Fisher, Waltham, Massachusetts, USA) [46]. The HPLC and MS conditions and parameter settings were established as previously described [47]. The raw data generated by HPLC-MS/MS were processed using the Compound Discoverer 3.3 (CD3.3, Thermo Fisher) to perform peak alignment/picking and metabolite quantitation. The metabolites were subsequently annotated using the KEGG database. Principal component analysis (PCA) and partial least squares discriminant analysis (PLS-DA) were performed using metaX (https://bio.tools/metax, accessed on 20 February 2024) [48]. The metabolites with variable importance in projection (VIP) > 1, *p* < 0.05, and a fold-change of ≥2 or ≤0.5 were considered to be differentially accumulated [44]. 

### 4.3. Transcriptomic Analysis

Total RNA was extracted using a polysaccharide polyphenol plant total RNA extraction kit according to the instructions. After RNA quality assessment, mRNA purification, and mRNA fragmentation, cDNA libraries were constructed, which were then sequenced on the Illumina NovaSeq platform, and raw reads were generated. The Trimmomatic v0.39 was used to filter the raw reads and generate clean data [49], which were then assembled de novo into unigenes using the Trinity 2.6.6 software [50]. Functional annotations of the unigenes were based on the Nr, Nt, Pfam, Swiss-Prot, KO, GO, and COG databases [51]. 

Gene expression was calculated using RSEM v1.3.1 [52]. The differentially expressed genes (DEGs) were identified using DEseq2, with screened criteria as the fold-change  ≥  2 and the corrected *p* value  <  0.05 [53]. Pearson’s correlation analysis was used to test the reliability of the samples. For functional prediction, the DEGs were subjected to GO and KEGG pathway annotation; GOseq 1.32.0 and KOBAS v3.0 were used for the GO and KEGG enrichment analyses of the DEGs [54,55]. The enrichment results of the GO and KEGG analyses were considered significant with the corrected *p* value < 0.05. Venn diagrams and bubble maps were constructed using the NovoMagic platform (https://magic.novogene.com/customer/main#/Login-New, accessed on 16 March 2024).

### 4.4. Transcriptional Regulatory Network Analysis

The identification and classification of the TFs were based on PlnTFDB [56] and PlantTFDB [57]. The DETFs were further screened. Cytoscape software version 3.6.1 was used to construct transcriptional regulatory networks for the DETFs and DAMs. Significant regulatory relationships between the DETFs and DAMs were determined by a Pearson correlation coefficient > 0.9 or <−0.8 and a *p* value < 0.05.

### 4.5. Validation of Gene Expression from Transcriptome Data

For real-time quantitative PCR (qRT-PCR), RNA samples were extracted from the ‘Sorbonne’ and ‘Tresor’ leaves (24 hpi) using an RNA extraction kit (Vazyme, Nanjing, China). Total RNA samples (500 ng) were transcribed into cDNA using a reverse transcription kit (Accurate Biology, Hunan, China). The SYBR Green Premix qPCR Kit (Accurate Biology, Hunan, Changsha, China) and the QuantStudio 6 Flex Real-Time PCR System (Thermo Fisher, Waltham, MA, USA) were used to analyze the expression of the candidate genes, as described in Figure 7. The experiments were performed using the following operating parameters: initial denaturation of the samples at 95 °C for 30 s, followed by 40 amplification cycles. Each cycle consisted of denaturation at 95 °C for 5 s and annealing at 60 °C for 30 s. The final stage of dissociation was 95 °C for 15 s, 65 °C for 1 min and 95 °C for 15 s. The specific information regarding the primer sequences can be found in Supplemental Table S6. The *EF1* gene was chosen to serve as the reference gene, in accordance with a previous study of the lily [18]. The relative expression levels of the target genes were calculated using the 2^−ΔΔCt^ method [58]. Each sample underwent three biological replicates and three technical replicates.

### 4.6. Plate Inhibition Assay of B. elliptica

Growth inhibition assays of *B. elliptica* were conducted on culture plates according to a previous study on *F. oxysporum* [6]. After pre-culturing *B. elliptica* on PDA, the fungal colony diameter was 2 cm; four sterile paper disks were evenly placed. The inhibitory effects of the phenylpropanes (hesperetin, ferulic acid, eriodictyol, and sinapyl alcohol) on *B. elliptica* mycelial growth were examined in separate plates. More specifically, the following reagents were added to the disks, respectively: 40 µL hesperetin solution (5, 10, and 15 mg·mL^−1^); 40 µL ferulic acid solution (5, 10, and 15 mg·mL^−1^); 40 µL eriodictyol solution (5, 10, and 15 mg·mL^−1^) or 40 µL sinapyl alcohol solution (5, 10, and 15 mg·mL^−1^); 40 µL DMSO (dimethyl sulfoxide) was added as the control solvent. The *B. elliptica* growth was recorded after incubation at 25 °C for 2 d using a digital camera (Canon EOS200d, Tokyo, Japan); then, Photoshop 7.0 was used to analyze the fungal growth area under the various solution treatments. The antifungal assays of these phenylpropanes were repeated three times.

### 4.7. Statistical Analysis

Each experiment was conducted three times, separately. The results were represented as the mean ± standard deviation (SD) of the biological triplicates. The Student’s *t*-test or Duncan’s multiple range test was employed in the analysis of the data; * *p* < 0.05 and ** *p* < 0.01 denoted the significance criterion.

## 5. Conclusions

To clarify the resistance mechanisms of the lily against *B. elliptica*, transcriptomic, and metabolomic analyses were conducted to show the differences in gene expression and metabolite biosynthesis between the resistant and susceptible lily cultivars. The integrated transcriptomics and metabolomics analyses revealed the critical roles of phenylpropanoid metabolism in protecting the lily from *B. elliptica*. The DAMs (eriodictyol, hesperetin, ferulic acid, and sinapyl alcohol) and DEGs (*F3′M*, *COMT*, and *CAD*) in the phenylpropanoid biosynthesis pathway were closely related to gray mold resistance formation, and these phenylpropanes could be the potential marker metabolites for gray mold resistance in the lily. Moreover, members of the AP2/ERF, WRKY, Trihelix, and MADS-M-type families positively regulated the biosynthesis of resistance-related phenylpropanes in the lily. The further transcriptional regulation study of the genes associated with phenylpropanoid biosynthesis could help us to obtain a better understanding of the differential defense mechanisms between resistant and susceptible lily cultivars.

## Data Availability

Data are contained within the article and Supplementary Materials.

## Supplementary Materials

The following supporting information can be downloaded at: https://www.mdpi.com/article/10.3390/ijms252011068/s1.
