# Supplementary material for: The Critical Role of Phenylpropanoid Biosynthesis Pathway in Lily Resistance Against Gray Mold
Source: Int J Mol Sci. 2024 Oct 15;25(20):11068. doi: 10.3390/ijms252011068 (PMC11507431; doi:10.3390/ijms252011068)
Supplement: Supplementary file 1 [file ijms-25-11068-s001.zip › ijms-3235230-supplementary.pdf]

International Journal of
Molecular Sciences

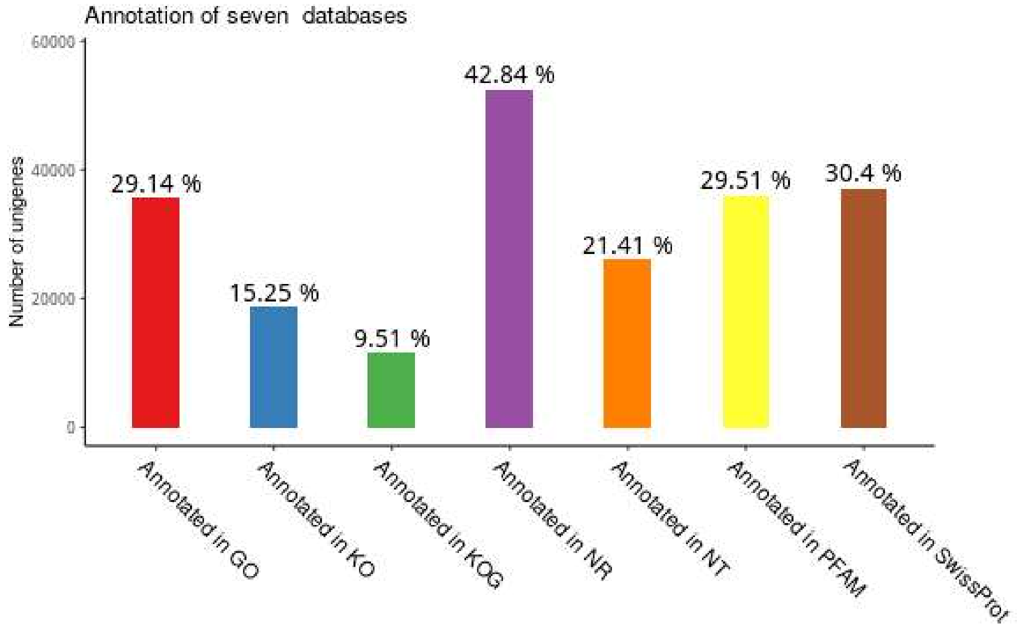

Figure S1. Annotation of the lily unigenes in databases.

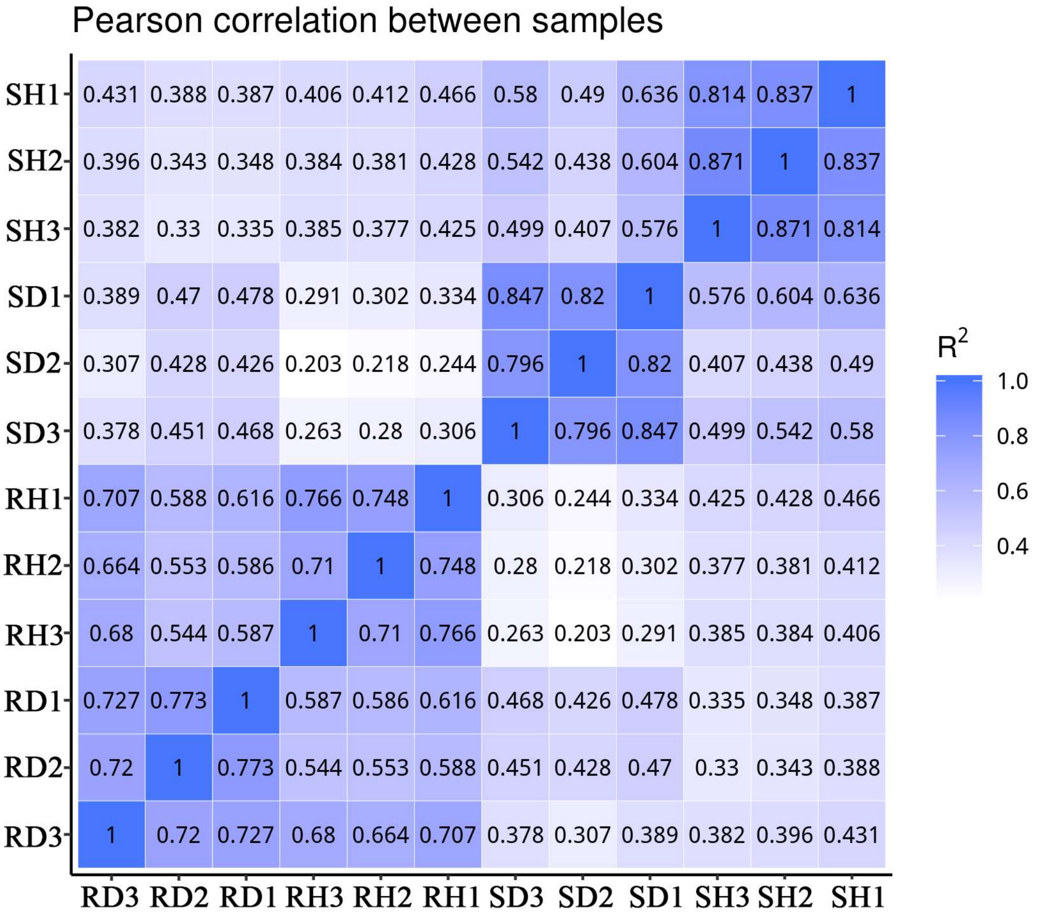

Figure S2. Repeatability evaluation of transcriptome data from lily after using Pearson's correlation analysis.

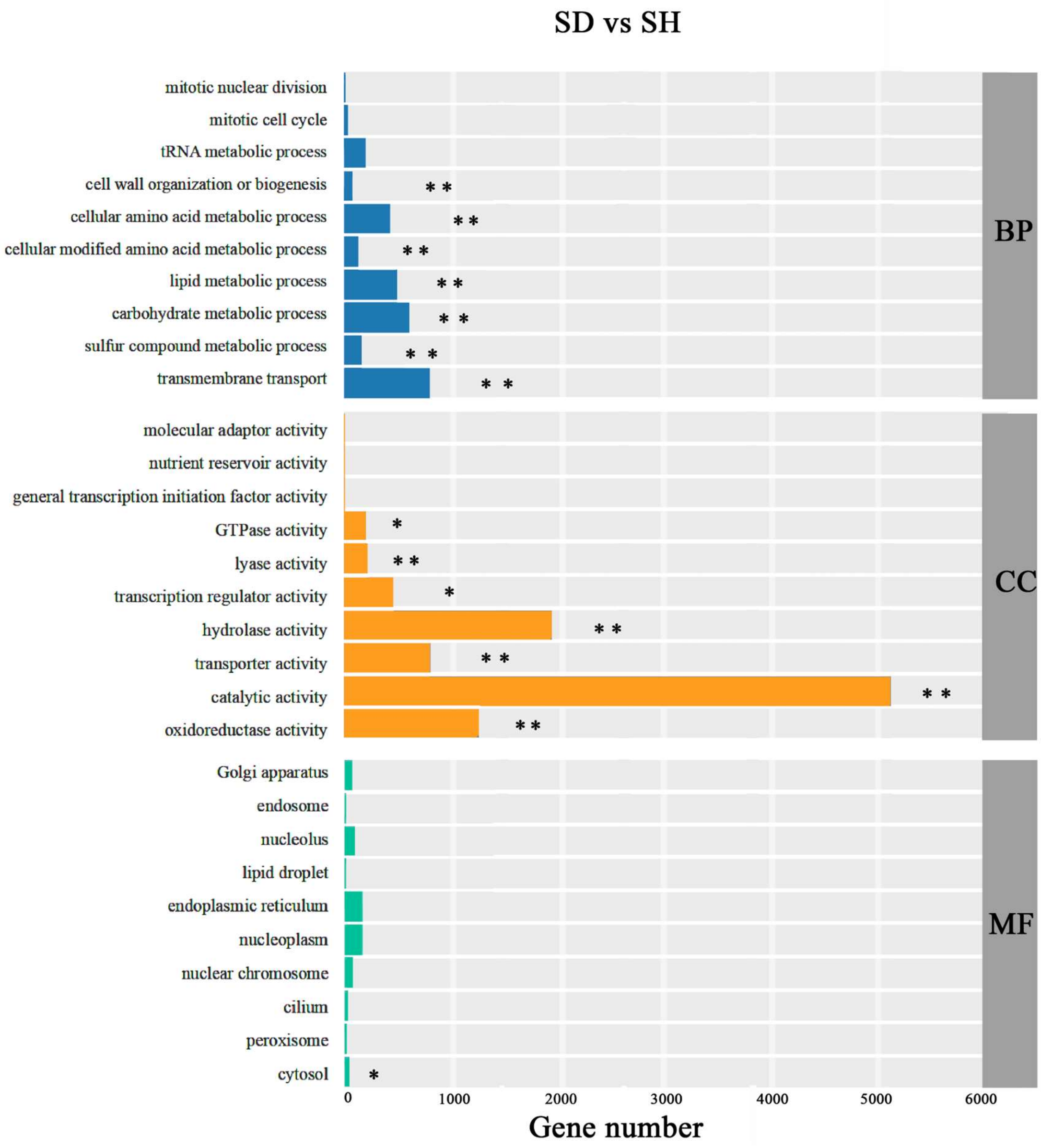

Figure S3. GO enrichment analysis of the DEGs detected in *B. elliptica*-infected 'Tresor' compared with its control. * $p < 0.05$, ** $p < 0.01$.

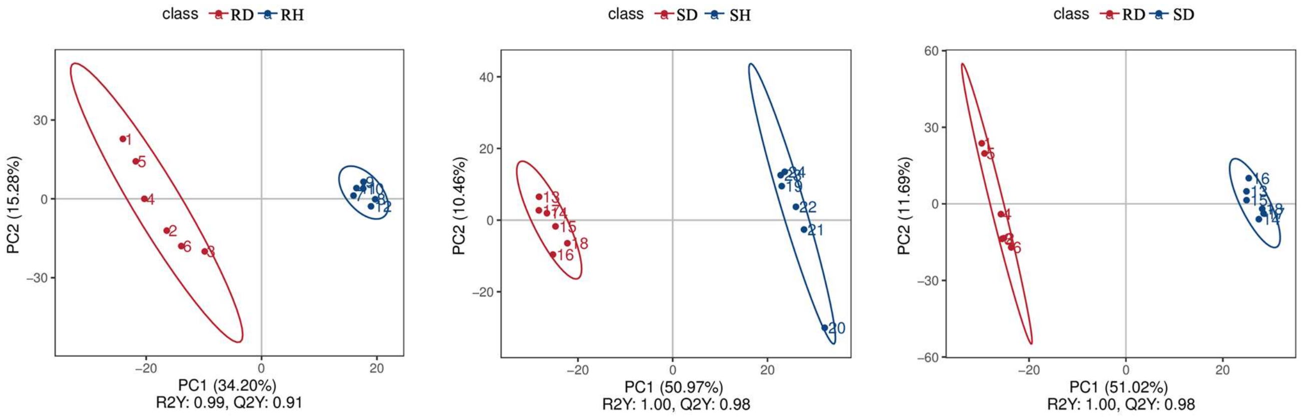

Figure S4. Partial least squares discriminant analysis (PLS-DA) for the samples based on the identified metabolites.

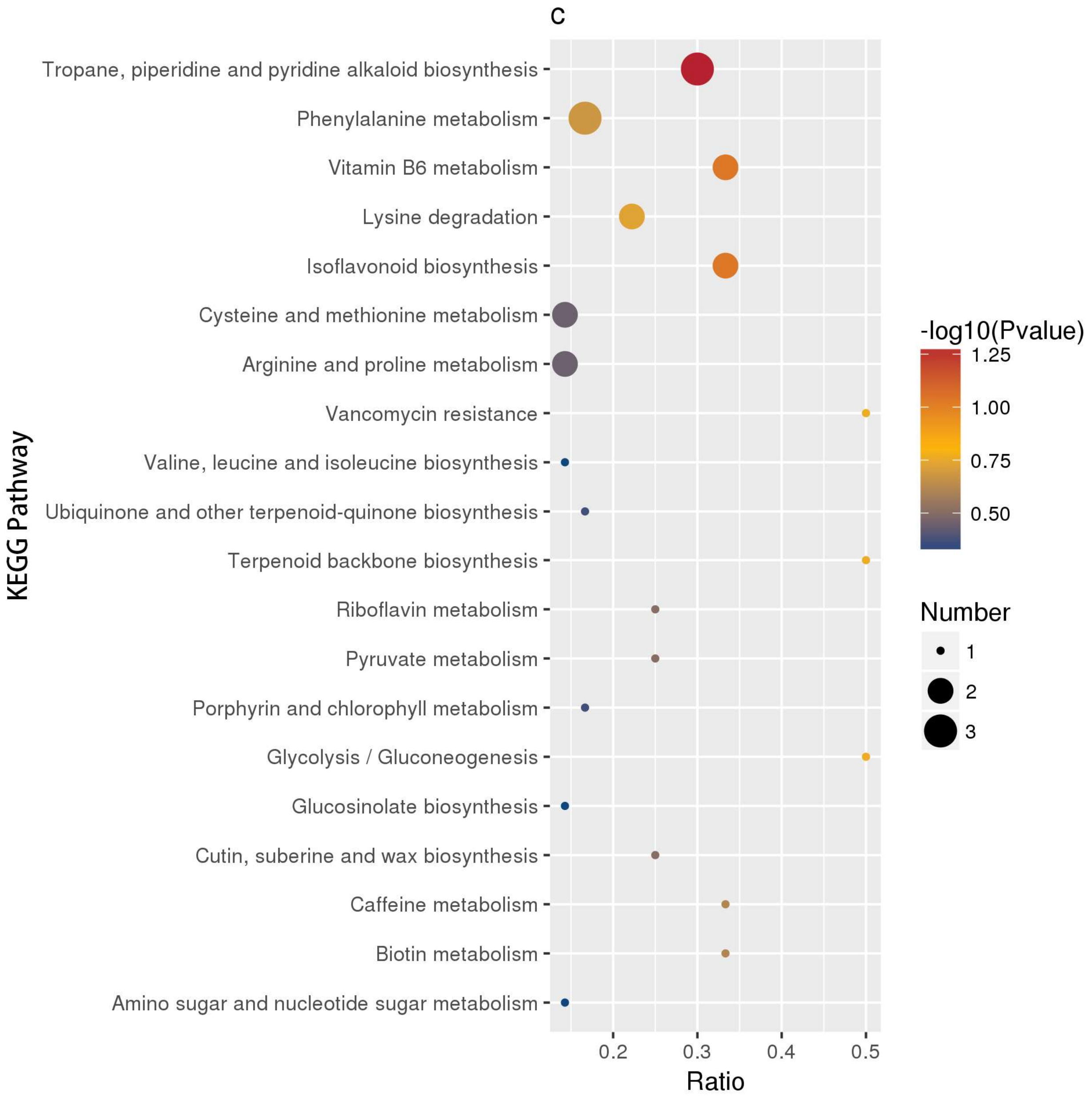

Figure S5. KEGG enrichment analysis for the 179 common DAMs.

Disclaimer/Publisher's Note: The statements, opinions and data contained in all publications are solely those of the individual author(s) and contributor(s) and not of MDPI and/or the editor(s). MDPI and/or the editor(s) disclaim responsibility for any injury to people or property resulting from any ideas, methods, instructions or products referred to in the content.
